# Impact of pharmacist-led home medicines review services on drug-related problems among the elderly population: a systematic review

**DOI:** 10.4178/epih.e2019020

**Published:** 2019-05-17

**Authors:** Sai Krishna Gudi, Ananth Kashyap, Manik Chhabra, Muhammed Rashid, Komal Krishna Tiwari

**Affiliations:** 1 Rady Faculty of Health Sciences, University of Manitoba College of Pharmacy, Winnipeg, Canada; 2 Department of Pharmacy Practice, Sarada Vilas College of Pharmacy, Mysuru, India; 3 Department of Pharmacy Practice, Indo-Soviet Friendship College of Pharmacy, Moga, India; 4 Department of Pharmacy Practice, Sri Adichunchanagiri College of Pharmacy, Adichunchanagiri University, Bala Gangadharanatha Nagara, Karnataka, India; 5 Department of Physiotherapy and Rehabilitation Sciences, Jagadguru Sri Shivarathreeshwara, College of Physiotherapy, Rajiv Gandhi University of Health Sciences, Karnataka, India

**Keywords:** Pharmacists, Drug-related side effects and adverse reactions, Drug interactions, Aged, Frail elderly

## Abstract

**OBJECTIVES:**

To address and elucidate the impact of pharmacist-led home medicines review (HMR) services on identifying drug-related problems (DRPs) among the elderly population in home care settings.

**METHODS:**

A comprehensive systematic search was performed using electronic scientific databases such as PubMed, Scopus, Embase, and Web of Science for studies published between January 1, 2008 and December 31, 2018, pertaining to HMR services by pharmacists for identifying DRPs.

**RESULTS:**

In total, 4,292 studies were retrieved from the searches, of which 24 were excluded as duplicates. Titles and abstracts were screened for the remaining 4,268 studies, of which 4,239 were excluded due to the extraneous nature of the titles and/or abstracts. Subsequently, 29 full-text articles were assessed, and 19 were removed for lacking the outcome of interest and/or not satisfying the study’s inclusion criteria. Finally, 10 studies were included in the review; however, publication bias was not assessed, which is a limitation of this study. In all studies, pharmacists identified a highly significant amount of DRPs through HMR services. The most common types of DRPs were potential drug-drug interactions, serious adverse drug reactions, need for an additional drug, inappropriate medication use, non-adherence, untreated indications, excessive doses, and usage of expired medications.

**CONCLUSIONS:**

HMR is a novel extended role played by pharmacists. The efficiency of such programs in identifying and resolving DRPs could minimize patients’ health-related costs and burden, thereby enhancing the quality of life and well-being among the elderly.

## INTRODUCTION

In countries with numerous and diverse populations, chronic diseases such as hypertension, diabetes, arthritis, and heart disease are common, especially among geriatric individuals; as a result, polypharmacy is a frequently occurring phenomenon in the elderly. Polypharmacy may give rise to various drug-related problems (DRPs), such as drug-drug interactions, adverse drug reactions (ADRs), medication errors, and drug-food interactions, which could eventually reduce levels of medication adherence [1]. At times, elderly patients might not take their medications as prescribed, which could evolve into altering the dose, frequency, or terminating the medication itself. The reasons for this may vary from simple logistical misunderstandings of factors such as the appropriate timing and dosage to a deeper misunderstanding of the purpose of a medication. Thus, inappropriate medication use among this vulnerable population is a major health concern [2]. ADRs are a major burden to patients, as shown by the fact that they are considered the fifth most common cause of death amongst hospitalized patients, with a notable morbidity rate of 5.6% in India. Therefore, several studies have been conducted to develop strategies for minimizing and preventing DRPs, including home medicines review (HMR) programs [3,4].

In 2001, the Australian government initiated the first HMR program, defined as a consumer-focused, structured, and collaborative health care service in the community setting to promote better medication adherence [5]. It is a team-based approach that involves a clinician, pharmacist, and consumer to optimize the quality use of medicines and to improve consumers’ understanding of their medications. HMR starts with a referral by a clinician, which enables the pharmacist to visit the patient’s home in order to review the current medication therapy of that particular patient, to check for potential DRPs, and to resolve them in consultation with the responsible clinician [1,2]. The essential goal of the HMR program is to reduce the DRPs that are derived from inappropriate use of medicines, as doing so could enhance patients’ medication adherence [6]. In this review, we sought to critically inspect studies of the effects of pharmacist-initiated HMR programs on identifying and mitigating DRPs.

## MATERIALS AND METHODS

### Data sources and search strategy

A comprehensive search was performed of PubMed, Scopus, Embase, and Web of Science for peer-reviewed, full-text articles published in the English language between January 1, 2008 and December 31, 2018. Relevant keywords such as “HMR,” “home medicines review,” “drug related problems,” “pharmacist,” and “elderly” were searched in diverse combinations with Medical Subject Headings (MeSH) terms by using Boolean operators to identify all relevant studies. The detailed search strategy interpretation using PubMed was as follows: (“HMR” [All Fields] OR “home medicines review” [All Fields]) AND “drug-related problems” [All Fields] AND “pharmacists” [MeSH Terms] OR “pharmacists” [All Fields] AND (“aged” [MeSH Terms] OR “aged” [All Fields] OR “elderly” [All Fields]) AND (“2008/01/01” [PDAT]: “2018/12/31” [PDAT]). Any further missing publications were searched by checking the references of the included studies. ProQuest, Google Scholar, and Open Grey were searched for the grey literature.

### Study selection and data extraction

Three reviewers (SKG, AK, and MC) independently screened the title and abstract of each article, and the potentially eligible full-texts of relevant abstracts were obtained and screened to identify articles of interest based on the study’s inclusion criteria, which were studies (prospective, retrospective, cross-sectional, or randomized) evaluating the impact of pharmacist-led HMR services on identifying DRPs among the elderly population. Articles were excluded if the outcome data were not reported in enough detail, the participants were not elderly, and the studies were conducted elsewhere from home or home care facilities; additionally, duplicate publications, literature reviews, conference abstracts, studies with no pharmacist involvement, and editorials/letters to the editor were excluded. The retrieved studies were imported into the Rayyan software [7] to remove duplicates and to review studies based on the inclusion and exclusion criteria. From each included study, the following data were extracted: author name(s), publication year, country, study design, sample size, mean age of the participants, key findings, and the summary. Any disagreements amongst the researchers regarding the inclusion of the studies were resolved through consensus, and a priori protocol was developed, and can be found in the Supplementary Material 1.

### Risk of bias and quality assessment

The risk of bias and methodological quality of each included study were assessed by 2 independent reviewers (AK and SKG) using the Standard Quality Assessment Criteria for Evaluating Primary Research Papers from a Variety of Fields [8], a 14-item measurement tool used to assess the methodological quality of the studies in a systematic review. Each item/question was scored as 2 (if the response was ‘yes’), 1 (if the response was ‘partial’), or 0 (if the response was ‘no’). Questions that were not applicable to a particular study were marked as ‘n/a’ and were excluded from the calculation of the summary score, which was calculated for each paper by summing the total score obtained for all items and dividing it by the total possible score. A higher summary score indicated a lower risk of bias and better study quality. Disagreements were resolved by discussions or by a third reviewer.

### Outcome assessment

The outcome of interest of this review was DRPs, which were assessed in terms of their frequency, type, and nature as described in each included study. DRPs were defined as an event or circumstance involving drug therapy that potentially interfered with desired health outcomes.

## RESULTS

**Figure 1.**
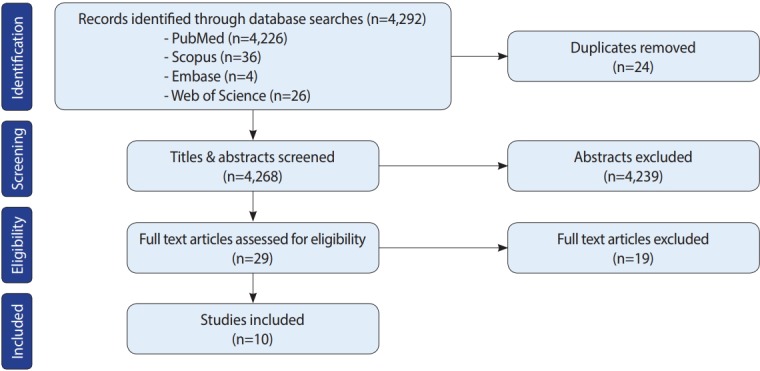
Preferred Reporting Items for System reviews and Meta-Analyses (PRISMA) flow-chart depicting the study selection process.

Initially, 4,292 studies were retrieved from the search, of which 24 were excluded as duplicates. Titles and abstracts were screened for the remaining 4,268 studies, of which 4,239 were excluded due to the extraneous nature of the titles and/or abstracts. Subsequently, 29 full-text articles were assessed, and 19 were removed for lacking the outcome of interest and/or not satisfying the study’s inclusion criteria. Finally, 10 studies were included in the review, as shown in detail in the PRISMA (Preferred Reporting Items for Systematic Reviews and Meta-Analyses) flow-chart in Figure 1.

### Characteristics of selected studies

The characteristics of the 10 included studies are described in Table 1. The plurality of the studies (4) were conducted in Australia [9-12], and one was conducted in each of the following countries: Sweden [13], India [14], Canada [15], Jordan [16], Germany [17], and Singapore [18]. Most of the studies utilized cross-sectional [13- 16] and retrospective study designs [9,11,12,18]. There was meaningful variation in the sample size across the included studies, ranging from 37 [17] to 1,720 [13]. In most of the studies, the mean age of the population was ≥ 65 years, except in 2 studies [14,16].

### Quality evaluation criteria

The quality of the studies was assessed using the Standard Quality Assessment Criteria for Evaluating Primary Research Papers from a Variety of Fields, which was developed by Kmet et al. [9]. The quality scores of most studies ranged from 80% to 100%, although 1 study [12] had the maximum score of 100% and 1 study [18] had a lower score (77%). Overall, the quality of the included studies was satisfactory. The quality scores of each study are presented in Table 2.

## DISCUSSION

HMR programs are emerging as one of the extended roles of community pharmacists in developed countries such as Australia, the USA, Canada, and various European countries [4,19,20]. Unfortunately, such programs have not been launched in most developing countries, such as India, for diverse reasons including the reluctance of general practitioners to follow recommendations made by pharmacists, a lack of awareness regarding HMR services among the public, patients’ conflicts of interest and privacy issues that affect their willingness to disclose their disease status and medication use, and linguistic and cultural diversity [5,6]. However, a few studies have investigated the influence of pharmacist-provided patient counseling services and found that such counseling services led to significant improvements in health outcomes in patients with chronic diseases [21-25]. The professional bodies in Australia have developed guidelines on HMR activities to assist community pharmacists in exercising professional judgment in individual health care circumstances and to promote the quality use of medicines to achieve better patient care. Diverse international studies of HMR services have reflected on optimizing medication use, minimizing DRPs, and improvising better health care outcomes in patients with chronic diseases and polypharmacy [26].

In addition to improving health care outcomes by fostering an understanding of one’s medicines and the ability to manage those medicines appropriately, HMR services also provide recommendations for general practitioners on any potential DRPs that may affect patient safety [27]. A study conducted by Dhillon et al. [6] on general practitioners’ perceptions of HMR programs confirmed that HMR improved general practitioners’ knowledge of the medicines that their patients were taking. A study conducted by Turner et al. [26] in Australia assessed the benefits of HMR and stated that pharmacists identified expired and unwanted over-the-counter medications with the patients during the comprehensive medication review [26]. Another study conducted by Gilbert et al. [3] to assess the usefulness of an HMR collaborative service that included 1,000 patients with 129 general practitioners and 63 pharmacists identified that a significant number (2,900) of DRPs were identified, of which 17% were wrong medication selection and 20% were poor medication adherence due to an inadequate knowledge of the drug on the part of consumers; in that study, the collaborative efforts of general practitioners and pharmacists helped to resolve 85% of the DRPs [3].

Besides identifying and resolving DRPs, HMR services provided by pharmacists could significantly decrease the rate of hospital readmission and its associated healthcare expenditures [18]. However, in a systematic review and meta-analysis conducted by Holland et al. [28], it was inferred that pharmacist-led medication review could reduce the number of drugs prescribed, and may improve patients’ drug-related knowledge and adherence behavior, but has no effect on mortality and morbidity. Furthermore, another study conducted by Pacini et al. [29] reported that HMR services provided by pharmacists did not reduce hospital admissions; instead, they observed only a minimal benefit in patients’ health-related quality of life that was not statistically significant.

In a quantitative survey of the views of HMR recipients conducted by Carter et al. [30], respondents reported the highest level of agreement that HMR would be of help in understanding more about their medications and rated the interpersonal skills of the visiting pharmacist as extremely high. In another study that attempted to assess the willingness of caregivers to assist their carerecipients with HMR, it was concluded that building expectations of HMR as an information resource among informal caregivers would likely increase the overall consumer demand for this service, which may ease the stress and burden of caregiving [31]. An Australian survey of consumers’ perspectives on HMR reported that those with the greatest need of the services were the least likely to receive HMR, and that the service recipients were well-satisfied with the HMR and recognized the benefits of the process [27]. However, patient involvement and cooperation are essential for them to receive a benefit from HMR services. In this review, we have sought to summarize and encapsulate the diverse findings of studies addressing the role of pharmacists in offering HMR services in community home care settings. Nonetheless, this study has certain limitations, including the inability to evaluate the results through a meta-analysis due to the small number of studies and the diverse outcome measures assessed in those studies, as well as the exclusion of studies without full-text access [32-35]. Furthermore, not appraising the risk of publication bias is a drawback of this study.

## CONCLUSION

If utilized appropriately, HMR services provided by pharmacists could assist patients in minimizing and/or averting DRPs to a significant extent, especially among the elderly. To prevent medication accidents and to improve adherence among patients, it is necessary to implement HMR services, and necessary measures should be taken by health regulatory bodies to increase awareness of HMR and to make use of existing HMR tools among the public. However, further robust research should be conducted to evaluate the effects of HMR programs on reducing hospital admissions and emergency visits.

## Figures and Tables

**Table 1.** Characteristics and key findings of the included studies

Author, year, country, study design, sample size, and mean age	Aim of the study	Intervention(s)	Outcome(s)	Results and key findings	Summary
Basheti et al. [16], 2011, Jordan, cross-sectional study, 167 patients, 58.9 yr	To assess the prevalence of TRPs and their types among chronic disease patients	HMR by pharmacists.	Prevalence and nature of TRPs	The mean number of disease conditions and number of medications per patient were found to be 4.1 ± 1.7 and 8.1 ± 2.7, respectively; The mean number of TRPs identified per patient through the HMR was 7.4 ± 2.8; Among the TRPs identified, 125 (74.9%) were incomplete drug therapy problems, 114 (68.3%) were untreated conditions, 101 (60.5%) were non-adherence to non-pharmacological therapy, 84 (50.3%) were inappropriate dosage regimens, 40 (23.9%) were adverse drug effects, and the fewest were potential drug interactions (n=17; 10.2%)	The study results demonstrated the integral role of pharmacists in identifying TRPs in Jordanian outpatients with chronic diseases visiting community pharmacies; Furthermore, patients were satisfied and accepted the HMR services offered by their community pharmacists, including the home visit aspect
Castelino et al. [9], 2009, Australia, retrospective cohort study, 224 patients, 74.65 yr	To assess the nature and extent of DRPs and the actions recommended by the pharmacists to resolve DRPs	HMR by pharmacists.	DRPs	Patients who were receiving HMR services were prescribed a mean (SD) number of 10.7 (3.8) medications; Pharmacists identified at least 1 DRP in 98% of the patients reviewed; Overall, the pharmacists identified a total of 1,110 DRPs, the most common (16%) being the need for an additional medicine; On average (SD), 4.9 (2.9) problems were identified per patient; Thirty-four percent of all the problems were related to the selection of a specific medicine, 24% to the medication dosing regimen and management issues, and 19% to patients’ knowledge and medication management skills	The study infers that a well-trained pharmacist with full access to the patients and their medical records and supporting resources could potentially enhance the quality use of medicines among the elderly population; It also suggests that most of the actions recommended by the pharmacists during the HMR process were consistent with the current literature
Chandrasekar et al. [14], 2017, India, cross-sectional study, 85 patients, 40-59 yr	To identify, prevent, and resolve potential MROs, optimize pharmacotherapy, and assist in achieving better health outcomes for patients at home	HMR by pharmacists	MRPs	Drug interactions were the main problem found in the majority of the prescriptions; Around 32% of the population experienced ADRs upon taking medications, and 64% of them did not use any over-the-counter drugs; In terms of knowledge gaps, multiple drug storage was the most critical error, while 34% of the patients were not aware of the name of a drug, 27% did not know the reason for taking a drug, and 27% were not aware of individual instructions given during pregnancy	This study suggested that qualified pharmacists can play a major role in improving the appropriateness of prescribing and preventing medication-related adverse events; Additionally, pharmacists in collaboration with general practitioners can optimize patients’ medications
Cheen et al. [18], 2016, Singapore, retrospective cohort study, 499 patients, >70 yr	To determine the impact of a pharmacist-provided HBMR program on readmissions in the elderly population	HBMR by a pharmacist	DRPs, readmission rate, ED visits, outpatient visits, and mortality	A total of 464 DRPs, corresponding to an average of about 5 DRPs per patient, were identified; Pharmacist-provided HBMR reduced readmissions by 26%, reduced ED visits by 20%, and increased outpatient visits by 16%; The most commonly identified DRPs were non-adherence (38.6%), untreated indication (22.4%), and overdosage (9.9%), and the pharmacists had resolved 36.4% of DRPs within 1 month of the home visit	This study suggested that pharmacist-led HBMR services led to significantly decreased readmissions and emergency visits among the elderly population; However, the mortality benefit was unclear, although there was a trend towards lower mortality among those who received HBMR
Elliott et al. [10], 2012, Australia, prospective randomized comparative study, 80 patients, 84 yr	To compare 3 different methods for promoting a pharmacist-led medication review for patients referred to an ACAT and to compare MRPs identified via ACAT usual care with those identified via pharmacist-led medication reviews	Comprehensive medication review by pharmacists	MRPs	Overall, 21 MRPs were identified via ACAT usual care: 5 (23.8%) were classified as high-risk, 10 (47.6%) as moderate-risk, 5 (23.8%) as low-risk, and 1 (4.8%) as insignificant; Pharmacists’ review of the ACAT files (without a pharmacist home visit) identified a further 164 potential MRPs; however, in the 40 patients who received an APHMR, 35 of 82 potential MRPs (42.7%) turned out not to be actual problems once further information was obtained from the patient; The APHMR identified 79 additional MRPs that were not identified from a review of the ACAT files; In total, 122 pharmacist-identified MRPs were included in APHMR reports to patients’ GPs; 94 of these were assessed as being associated with moderate, high, or extreme risk of an adverse event if not addressed	The study revealed that adding a pharmacist to the usual care assessment teams could significantly help in identifying and resolving MRPs; In addition, it was also inferred that home visits by a pharmacist can serve as a more efficient way for identifying MRPs than a routine medication review of the collected data; Furthermore, adding pharmacists to ACATs may provide a reliable and cost-effective method for delivering medication reviews, which reduce the risk of adverse events
Fiss et al. [17], 2010, Germany, prospective cohort study, 37 patients, 75.5 yr	To establish an interdisciplinary health professional network to systematically identify and evaluate DRPs in the patients’ homes, and to provide recommendations	Community-based HMR	DDIs	During a GP–supporting, community-based, e-health assisted, systemic intervention, 56 potential DDIs were identified, and 37 of the 112 drugs which caused potential interactions were attributed to OTC medication and food components; The mean number of drugs recorded per patient was 14.2; The evaluations of clinically relevant potential DDIs yielded relevant DDIs in 44.6% of the patients (n=25)	The study results suggested that a notable prevalence of DRPs was identified by a comprehensive HMR conducted by GP–supporting, community-based, e-health assisted, systemic intervention practice assistants in cooperation with local pharmacists
Gheewala et al. [12], 2014, Australia, retrospective cohort study, 847 patients, 84.9 yr	To investigate the number and nature of DRPs and recommendations made by pharmacists among residents of aged care facilities	Collaborative RMMR service by pharmacists	DRPs	Of the 847 included patients, the mean (SD) number of medications prescribed per resident was 11.2 (4.8); The pharmacists identified a total of 2,712 DRPs in 98% of the residents; The mean (SD) number of DRPs identified per resident was 3.2 (1.7); Of 3,054 recommendations made, 2,560 (83.8%) were accepted by the GP; The mean (SD) number of recommendations made per resident by the pharmacist was 3.6 (1.9) and mean (SD) number of recommendations accepted by the GP per resident was 3.0 (1.9)	The study suggested that the collaborative RMMR service with the help of an accredited pharmacist could significantly reduce DRPs among the residents of aged care facilities
Lenander et al. [13], 2018, Sweden, cross-sectional study, 1,720 patients, 87.5 yr	To evaluate the effect of medication reviews on total medication use and potentially inappropriate drug use among elderly patients, and to describe the occurrence and types of DRPs	Medication review by clinical pharmacists	DRPs	Of the 1,720 patients, 61% of them were on 10 or more drugs (range, 1-35); DRPs were identified in 84% of the patients, and a total of 3,868 DRPs were identified, giving a mean of 2.2 DRPs per patient; The most frequent types of DRPs (n = 3,868) identified were unnecessary drug therapy (39%), the wrong drug (20%), and an excessively high dose of medications (21%); Drug withdrawal was identified as the most common result	This study inferred that medication reviews performed in daily care by clinical pharmacists are one way to identify DRPs and to improve drug use among elderly patients; It also concluded that drug use is voluminous among elderly patients in home care and nursing home residents, and that additional drug therapy is a common problem
Nishtala et al. [11], 2009, Australia, retrospective cohort study, 500 patients, 84 yr	To investigate the number and nature of DRPs identified by accredited clinical pharmacists	Medication review by accredited clinical pharmacists.	DRPs	In a 500 randomly selected, de-identified medication reviews performed by 10 accredited clinical pharmacists over 6 months across 62 aged care homes, a total of 1,433 MRPs were identified in 480 residents; Potential DRPs were classified as a need for additional monitoring, risk of ADRs, and inappropriate choice of a drug; Among identified DRPs, alimentary, cardiovascular, central nervous system and respiratory drugs were most frequently implicated, accounting for more than 75% of the DRPs	The study concluded that clinical pharmacists have a potential role in identifying DRPs among older people living in aged care homes; Moreover, the recommendations made by pharmacists to minimize the risk of ADRs and to optimize drug choices were accepted and implemented by GPs
Papastergiou et al. [15], 2013, Canada, cross-sectional study, 43 patients, 77.4 yr	To identify and resolve the drug therapy problems of homebound patients	Pharmacist-directed HMR	Drug-therapy problems	The patients were taking a mean of 11.7 (range, 3-23) medications; Pharmacists identified a total of 62 drug therapy problems; The top 3 types of problems identified were non-compliance (40.3%), ADRs (20.9%) and additional therapy required (19.4%); Of the seniors, 44% were found to be using at least 1 medication on the Beers criteria list, whereas 7% were using 3 or more; Medications were removed from the homes of 58% of the patients, most commonly due to expiry of medication	The study concluded that pharmacists are among the most accessible front-line primary care practitioners and can provide care to home-bound patients; Pharmacist-directed HMRs offer an effective mechanism to address pharmacotherapy issues and could serve to minimize the inappropriate use of medication and health care costs

TRPs, treatment-related problems; HMR, home medications review; DRPs, drug-related problems; SD, standard deviation; ADRs, adverse drug reactions; HBMR, home-based medication review; ED, emergency department; ACAT, aged care assessment team; MRPs, medication-related problems; APHMR, ACAT-initiated pharmacist home medicines review; DDIs, drug-drug interactions; OTC, over-the-counter; GP, general practitioner; RMMR, residential medication management review.

**Table 2.** Quality evaluation of the included studies

Criteria	Study									
	[9]	[10]	[17]	[18]	[11]	[13]	[12]	[14]	[15]	[16]
Question/objective sufficiently described?	2	2	2	2	2	2	2	2	2	2
Study design evident and appropriate?	2	2	2	2	2	2	2	2	2	2
Method of subject/comparison group selection or source of information/input variables described and appropriate?	2	2	2	2	2	2	2	1	2	2
Subject (and comparison group, if applicable) characteristics sufficiently described?	2	2	2	2	2	2	2	1	2	2
If interventional and random allocation was possible, was it described?	N/A	2	N/A	N/A	N/A	N/A	N/A	N/A	N/A	N/A
If interventional and blinding of investigators was possible, was it reported?	N/A	0	N/A	N/A	N/A	N/A	N/A	N/A	N/A	N/A
If interventional and blinding of subjects was possible, was it reported?	N/A	2	N/A	N/A	N/A	N/A	N/A	N/A	N/A	N/A
Outcome and (if applicable) exposure measure(s) well defined and robust to measurement / misclassification bias? Means of assessment reported?	2	2	1	2	2	2	2	2	2	2
Sample size appropriate?	2	2	1	2	2	2	2	2	1	2
Analytic methods described/justified and appropriate?	2	2	2	2	2	2	2	2	1	2
Is some estimate of variance is reported for the main results?	0	2	0	2	2	0	0	0	0	0
Controlled for confounding?	N/A	0	1	1	N/A	N/A	N/A	N/A	N/A	N/A
Results reported in sufficient detail?	2	2	2	2	2	2	2	2	2	2
Conclusions supported by the results?	2	2	2	2	2	2	2	2	2	2
Maximum points	20	28	22	22	20	20	20	20	20	20
Total points	18	24	17	21	20	18	18	16	16	18
Summary score (%)	90	86	77	95	100	90	90	80	80	90

0, if the response is ‘no’; 1, if the response is ‘partial’; 2, if the response is ‘yes’; N/A, not applicable.
