# Supplementary material for: Impact of pharmacist-led home medicines review services on drug-related problems among the elderly population: a systematic review
Source: Epidemiol Health. 2019 May 17;41:e2019020. doi: 10.4178/epih.e2019020 (PMC6635662; doi:10.4178/epih.e2019020)
Supplement: Supplementary file 1 [file epih-41-e2019020-supplementary.pdf]

Impact of pharmacist-led home medicines review services on drug related problems among elderly population: a systematic review

Sai Krishna Gudi, Manik Chhabra, Muhammed Rashid, Ananth Kashyap, Komal Krishna Tiwari

Citation

Sai Krishna Gudi, Manik Chhabra, Muhammed Rashid, Ananth Kashyap, Komal Krishna Tiwari. Impact of pharmacist-led home medicines review services on drug related problems among elderly population: a systematic review. PROSPERO 2019 CRD42019123797 Available from: http://www.crd.york.ac.uk/PROSPERO/display_record.php?ID=CRD42019123797

Review question

Do Home Medicines Review (HMR) services offered by the Pharmacist play an important role in identifying the Drug Related Problems (DRPs) among elderly population?

Searches

The project will be carried-out based on the guidelines of systematic review using the Preferred Reporting Items for Systematic Reviews and Meta-Analyses (PRISMA) guidelines in order to identify the relevant articles. A literature search will be performed using the scientific data sources included PubMed, Scopus, EMBASE, Web of Science and Google Scholar. The search period includes the peer-reviewed full-text articles published in the English language between January 2008 to December 2018 will be searched (10 years). The key search terms that would be used are "Home Medicine Review", "Home Medication Review", "HMR", "Drug Related Problems", "Medication Related Problems", "Treatment Related Problems", "DRP", "Pharmacist", "Elderly", and "Aged".

Types of study to be included

The proposed review will include studies which are prospective, retrospective, cross-sectional, and randomized in nature. The study types such as, literature reviews, letter to editors, pilot studies, and short communications would be excluded from the review.

Condition or domain being studied

The domain being studied in this project would be an extended role of the pharmacist in providing medication review services at home care facilities of elderly population and the outcome measure would be identifying the frequency of drug related problems across the studies.

Participants/population

Studies either prospective, retrospective, or randomized in nature that evaluating the impact of pharmacists' medication review services in identifying the drug related problems as their one of the objectives with elderly population age greater than equal to 65 years in home care/nursing home facilities. Any studies which were conducted else where (like hospital settings) other than home settings, without involvement of the pharmacist, and not

assessing drug related problems as their objective would be excluded from the study. In addition, studies published in other languages apart from English, and studies which were review in nature such as, literature review, letter to the editor, pilot studies, and short communications are not considered for the review.

Intervention(s), exposure(s)

The intervention/exposure in this systematic review would be Home Medicine Review. HMR would be defined as the medication review services offered by the pharmacist upon referral from the general practitioner (GP) in a home setting with an intention to prevent and resolve drug related problems.

Comparator(s)/control

When it comes to this systematic review, it doesn't have any concern of having or not having a comparator/control group, as this review's major focus is to identify the frequency of DRPs rather than comparing with any control group. As mentioned, this review might also consider randomized controlled studies with control group as patients who had not received HMR services by the pharmacist.

Context
Studies which were performed at home/home care/nursing home settings which were conducted across the globe would be considered for the review.

Main outcome(s)
To summarize the available evidence on the effect of pharmacist-led HMR in identifying the DRPs among elderly patients in home settings. The specific outcome that the review looking at is the frequency/number of DRPs identified by pharmacist through HMR services. Furthermore, the investigated findings would be summarized narratively.

Additional outcome(s)
None.

Data extraction (selection and coding)
The screening of the title and abstract of each article, and the potentially eligible full-texts of relevant abstracts would be obtained and screened to identify articles based on the above mentioned inclusion criteria by three independent reviewers. From each study the following data will be extracted, author name(s), publication year, country, the total number of study participants, mean age of study participants, outcome measures, key findings, and summary. Any conflicts on inclusion will be resolved through consensus by the reviewers.

Risk of bias (quality) assessment
Risk of bias and quality assessment of the selected studies will be performed by using the NIH Quality Assessment Tool. Two independent reviewers will assess the risk of bias and quality of the included studies and any discrepancies will be resolved through consensus by all the project reviewers.

Strategy for data synthesis
The included studies will be categorized based on the design, and findings are compared and alternatively reported. The aggregate data (overall frequency of DRPs among individual studies) will be used in reporting the findings qualitatively. As this topic is novel and not many studies are conducted addressing this research question, and variance in the outcome across the existed studies, performing a quantitative synthesis or meta-analysis would not be possible. Of five researchers, three reviewers independently will screen the title & abstract of each article, and the potentially eligible full-texts of relevant abstracts will be imported into Rayyan software to remove the duplicates and review the studies based on the inclusion & exclusion criteria. Any disagreements among the researchers regarding the inclusion of the studies will be resolved through consensus. Risk of bias and methodological quality of each included study will be performed by two independent reviewers by using the NIH Quality Assessment Tool. Since it is a qualitative systematic review, statistical heterogeneity will not be an ideal approach to consider.

Analysis of subgroups or subsets
If the relevant data is available, analysis will be done on type of DRPs identified across the studies.

Contact details for further information
Sai Krishna Gudi
sknanu06@gmail.com

Organisational affiliation of the review
University of Manitoba

http://umanitoba.ca/faculties/health_sciences/pharmacy/

Review team members and their organisational affiliations
Dr Sai Krishna Gudi. University of Manitoba
Mr Manik Chhabra. Indo-Soviet Friendship College of Pharmacy
Mr Muhammed Rashid. Sri Adichunchanagiri College of Pharmacy
Mr Ananth Kashyap. Sarada Vilas College of Pharmacy
Dr Komal Krishna Tiwari. Rajiv Gandhi University of Health Sciences

Type and method of review
Narrative synthesis, Systematic review

Anticipated or actual start date
01 December 2018

Anticipated completion date
28 February 2019

Funding sources/sponsors
None.

Conflicts of interest

Language
English

Country
Canada, India

Stage of review
Review Ongoing

Subject index terms status
Subject indexing assigned by CRD

Subject index terms
Aged; Humans; Pharmaceutical Services; Pharmacists

Date of registration in PROSPERO
02 April 2019

Date of publication of this version
02 April 2019

Details of any existing review of the same topic by the same authors

Stage of review at time of this submission

Stage	Started	Completed
Preliminary searches	Yes	Yes
Piloting of the study selection process	Yes	Yes
Formal screening of search results against eligibility criteria	Yes	Yes
Data extraction	No	No
Risk of bias (quality) assessment	No	No
Data analysis	No	No

Versions
02 April 2019

faith and registered the review in PROSPERO. The registrant confirms that the information supplied for this submission is accurate and complete. CRD bears no responsibility or liability for the content of this registration record, any associated files or external websites.
